# Supplementary material for: Band power modulation through intracranial EEG stimulation and its cross-session consistency
Source: J Neural Eng. Author manuscript; Available in PMC 2022 Feb 1. (PMC7612301; doi:10.1088/1741-2552/abbecf)
Supplement: Supplementary [file EMS140916-supplement-Supplementary.pdf]

Supplementary material for:

Band power modulation through intracranial EEG stimulation and its cross-session consistency

Christoforos A Papasavvas, Gabrielle M Schroeder, Beate Diehl, Gerold Baier, Peter N Taylor, Yujiang Wang

Fig. S1 : Organization of stimulation trials and the pre-post segments around the pulse trains and during the inter-stimulus intervals. Stimulation trials were typically delivered in groups of 3. Stimulation pre/post segments were extracted around each trial. Baseline pre/post segments (3 pairs) were extracted from the middle of inter-stimulus intervals that are at least 20s long. The gap between pre and post during baseline is equal to the gap between the pre and post segments around the trials (black arrows).

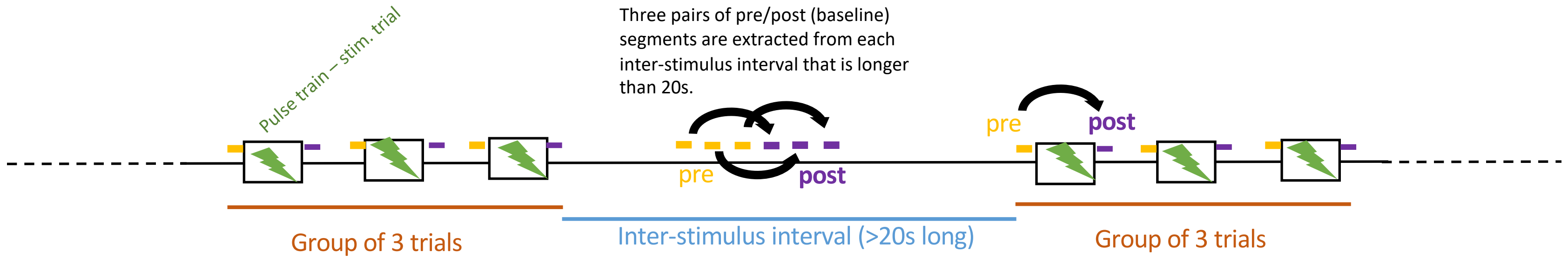

Fig. S2-1: Distributions of minimum and maximum effects U on delta band for both baseline (green) and stimulation (yellow). Each dot represents one session. The lower panels show the distribution of paired differences (pairs between baseline and stimulation within a session).

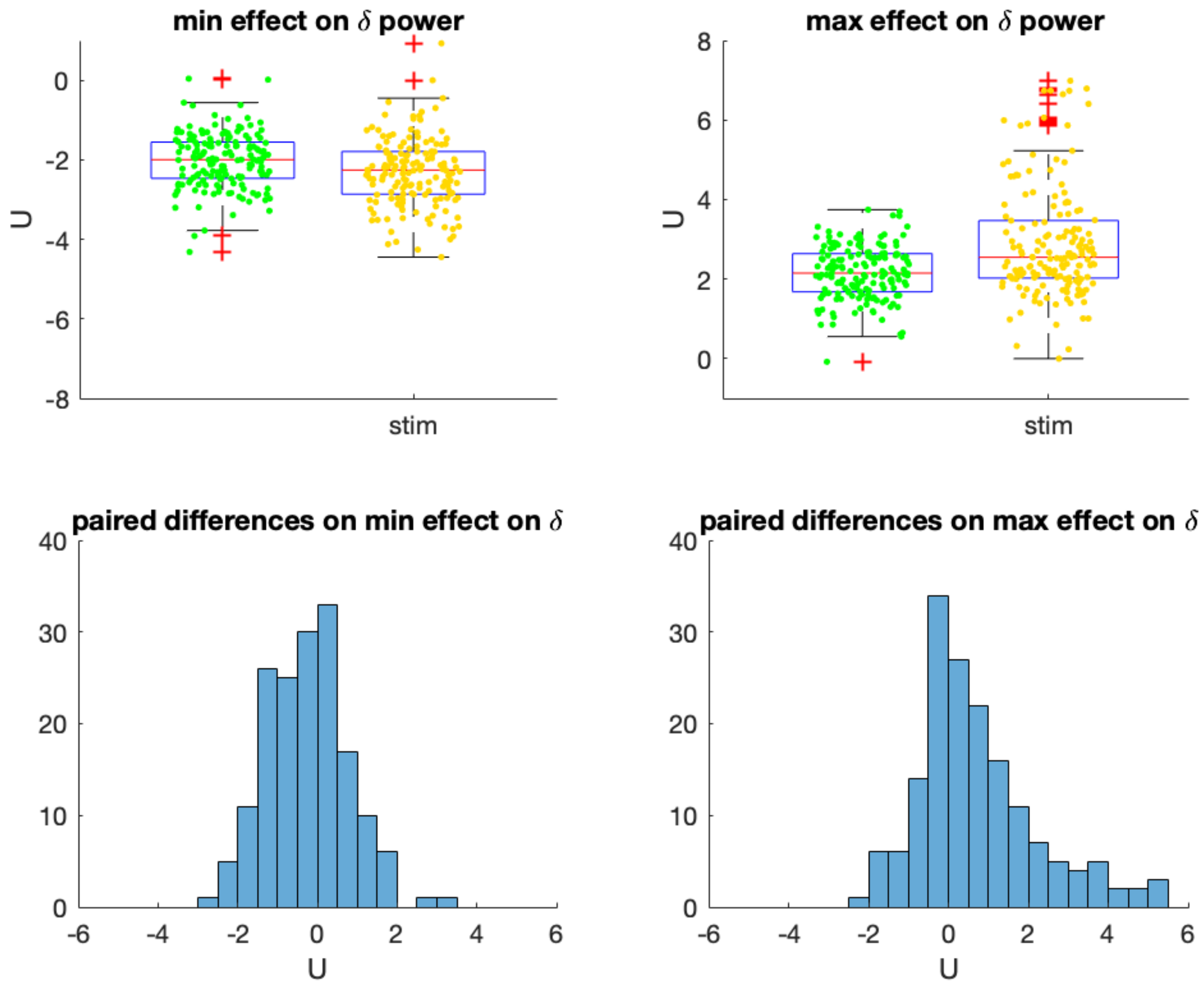

Fig. S2-2: Distributions of minimum and maximum effects U on theta band for both baseline (green) and stimulation (yellow). Each dot represents one session. The lower panels show the distribution of paired differences (pairs between baseline and stimulation within a session).

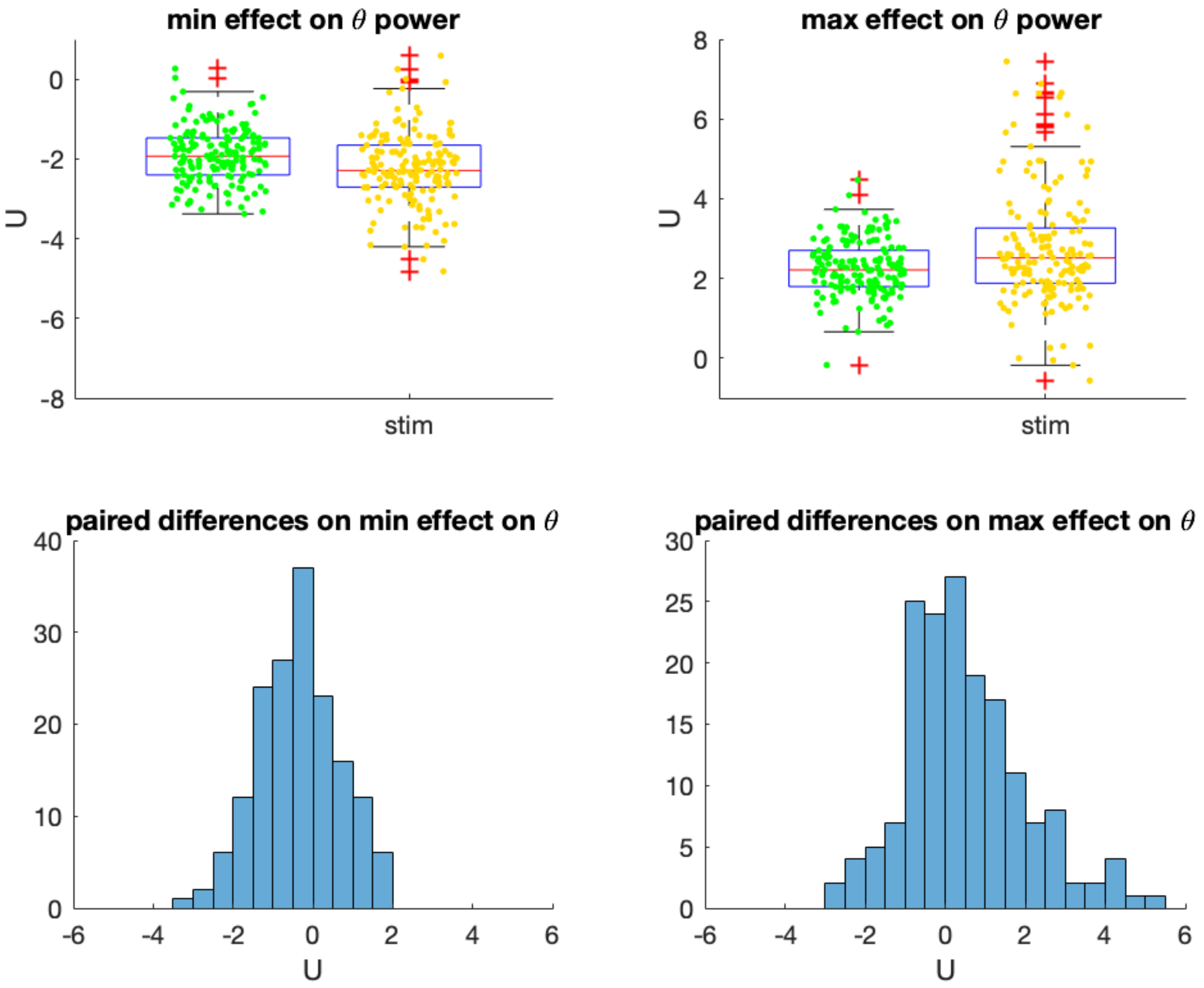

This is the same as in Fig. 3

Fig. S2-3: Distributions of minimum and maximum effects U on alpha band for both baseline (green) and stimulation (yellow). Each dot represents one session. The lower panels show the distribution of paired differences (pairs between baseline and stimulation within a session).

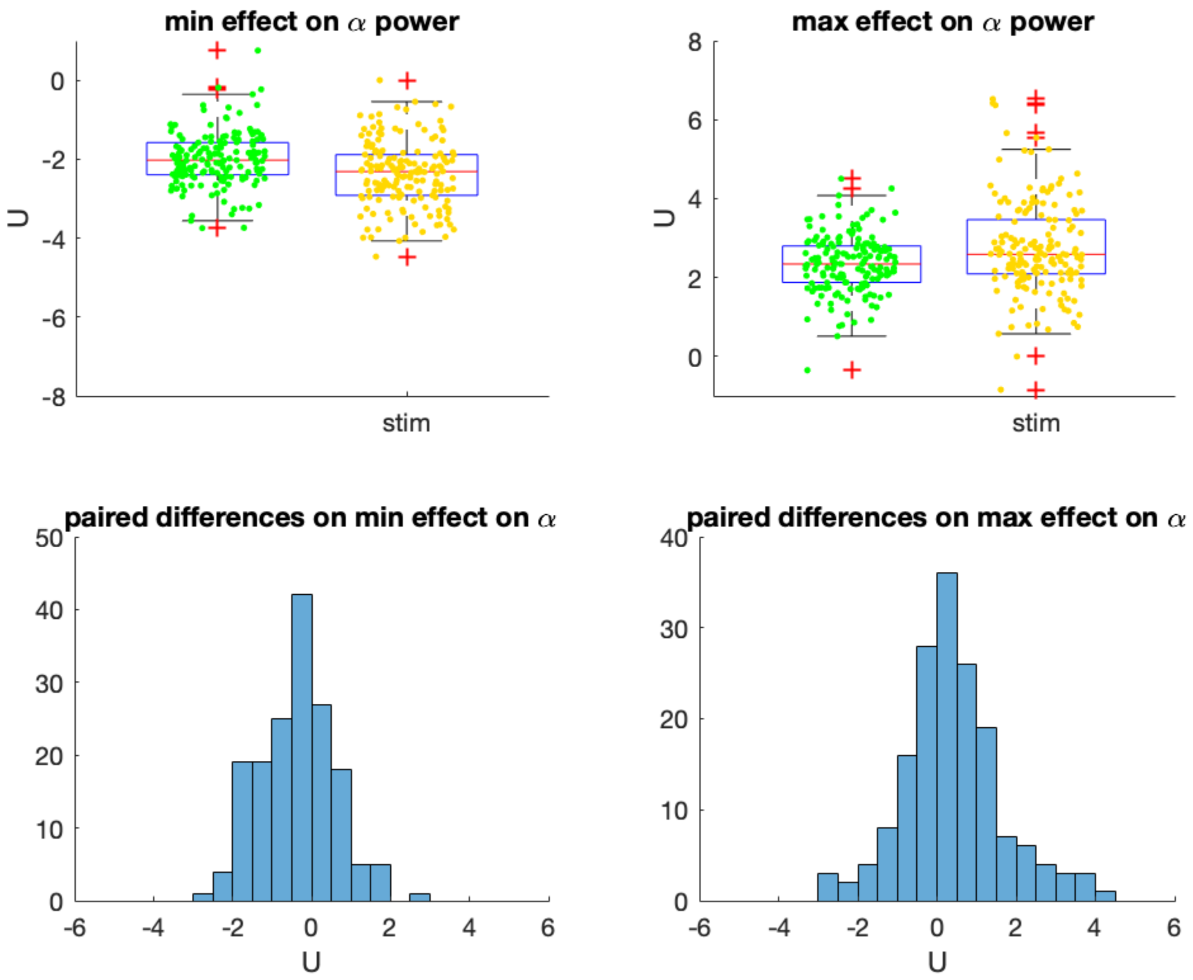

Fig. S2-4: Distributions of minimum and maximum effects U on beta band for both baseline (green) and stimulation (yellow). Each dot represents one session. The lower panels show the distribution of paired differences (pairs between baseline and stimulation within a session).

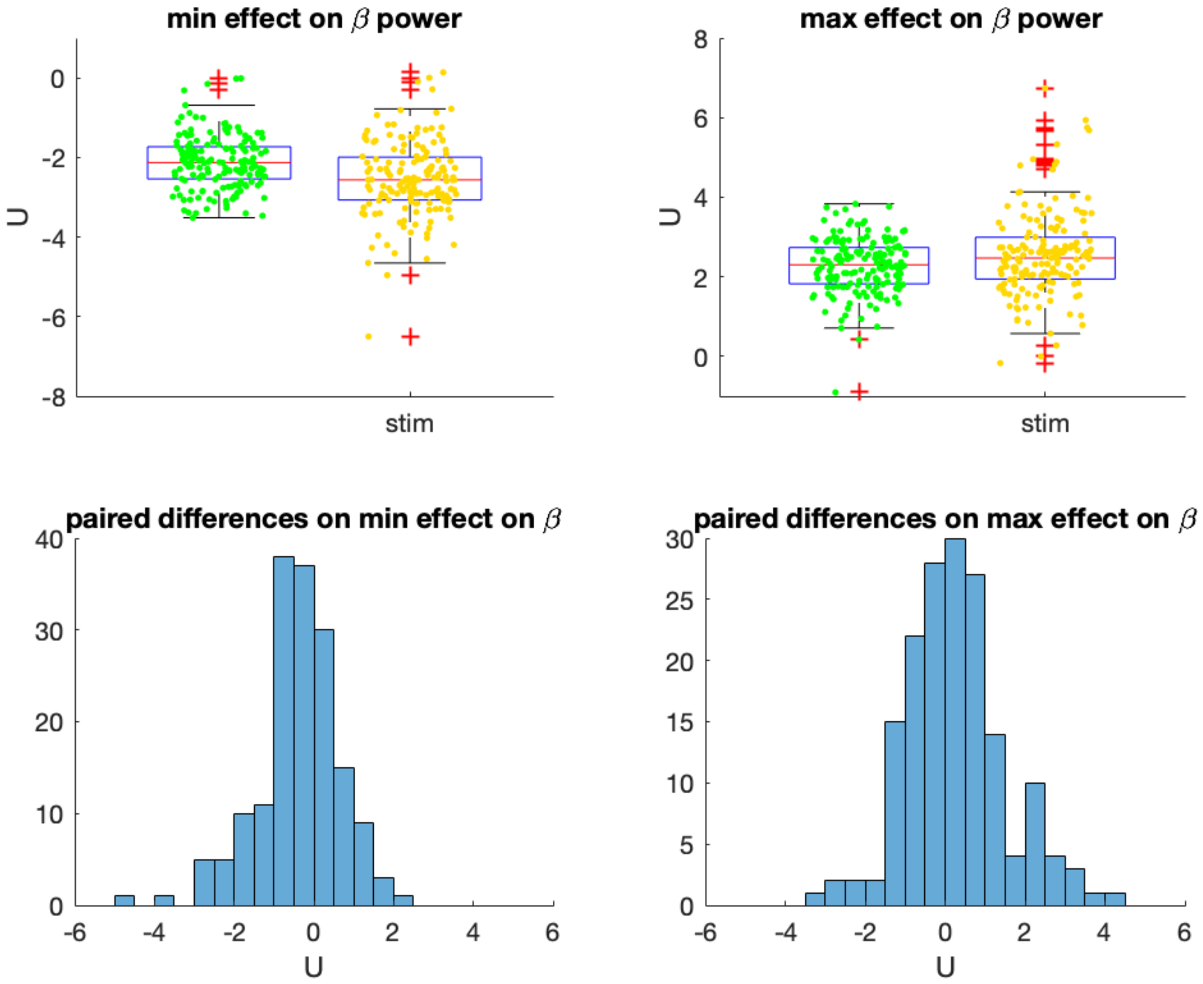

Fig. S2-5: Distributions of minimum and maximum effects U on gamma band for both baseline (green) and stimulation (yellow). Each dot represents one session. The lower panels show the distribution of paired differences (pairs between baseline and stimulation within a session).

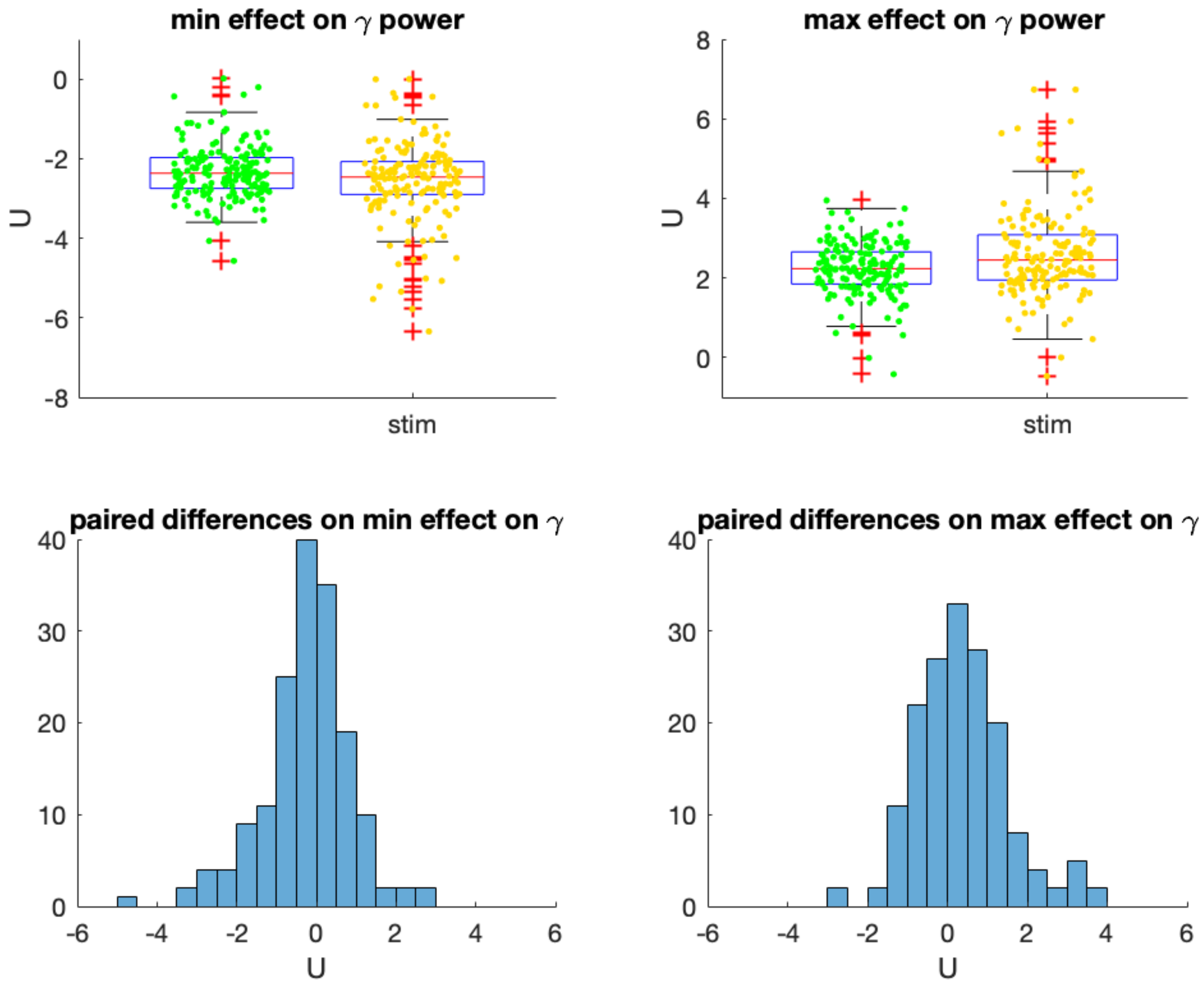

Fig. S3: Band specific results on the relation between stimulation effect (represented with extremes: min and max) and the stimulation amplitude.

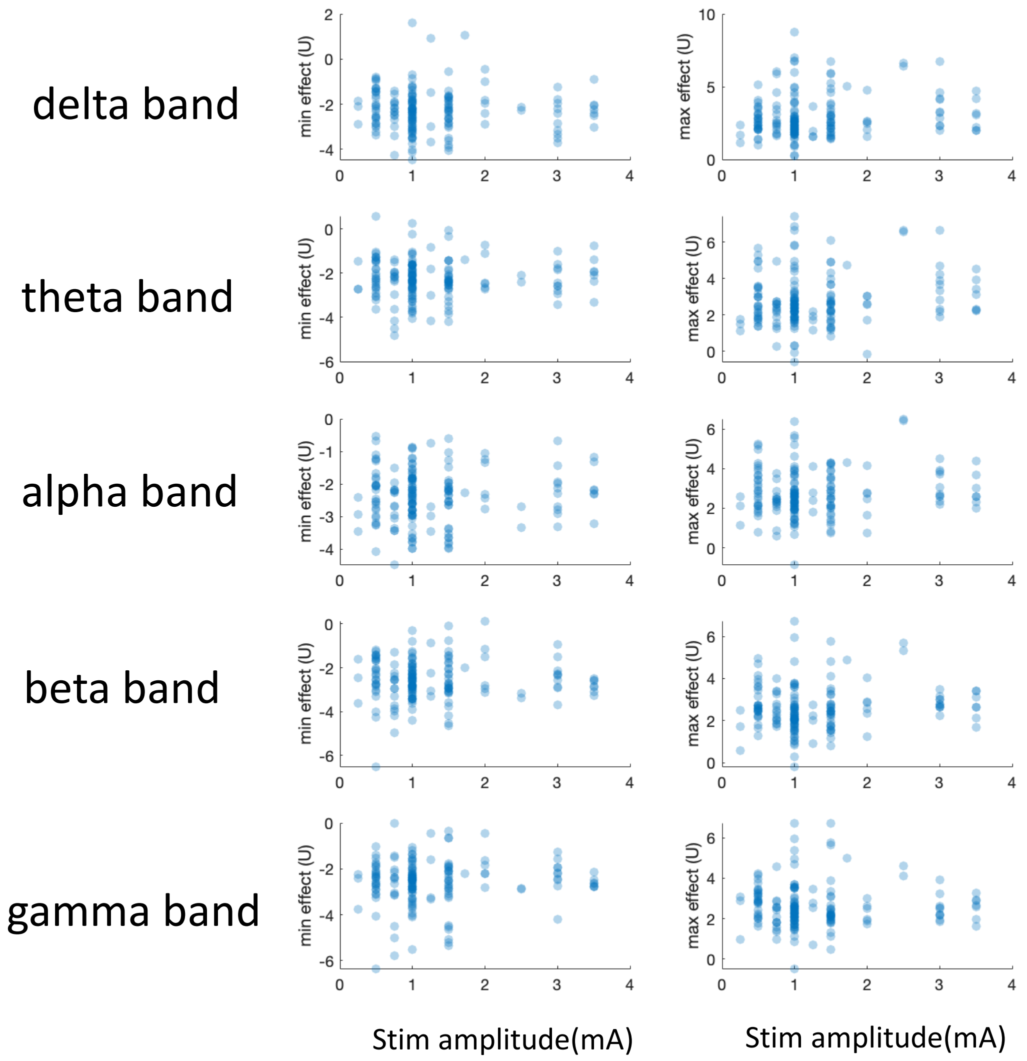

Significantly lower variance only in min effect in gamma band for ampl>1.5mA. Applied Brown-Forsythe's Test on two groups: ampl <=1.5 and ampl> 1.5

Fig. S4: Predictive power of average max effect on consistency - contributions of the different frequency bands. The average max effect on theta and alpha has the highest predictive power over max consistency coefficient.

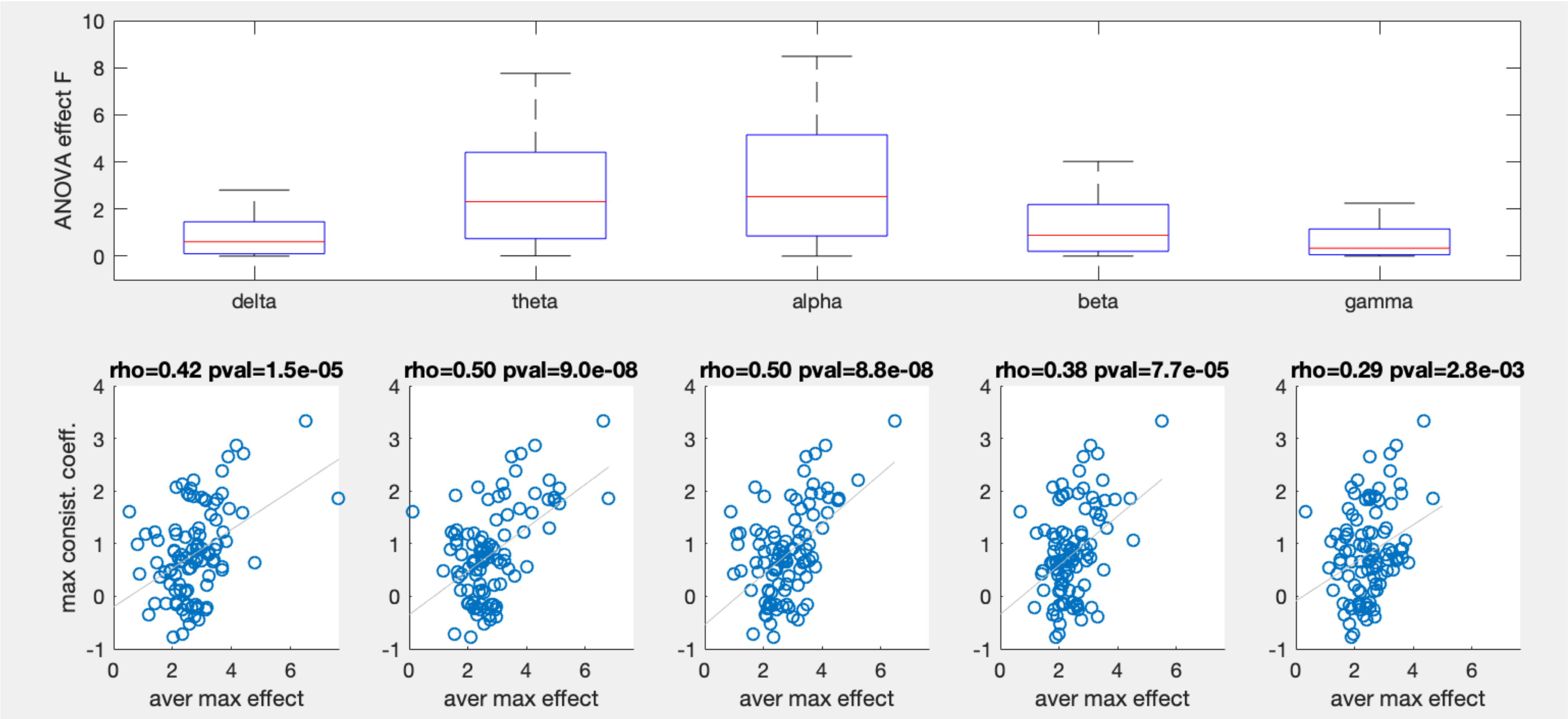

Fig. S5: Band specific results on the relation between stim depth and effect U (represented by the extremes min and max).

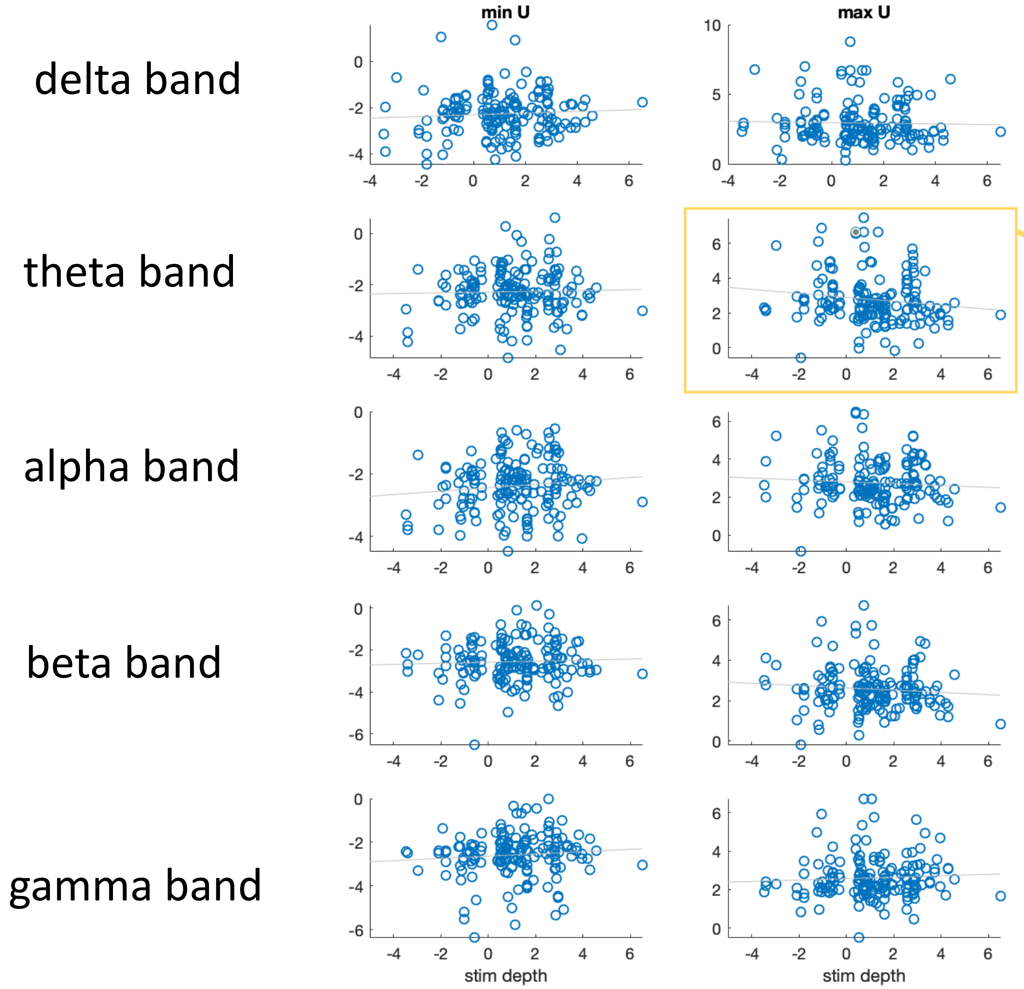

The most significant relation is a weak anti-correlation between max effect on theta and stim depth
r=-0.138   p=0.076

Fig. S6-1: All stim locations – Colours indicate the maxima and minima effects elicited (in other locations) by the stimulation - Delta band

Locations marked are the midpoints between anode and cathode

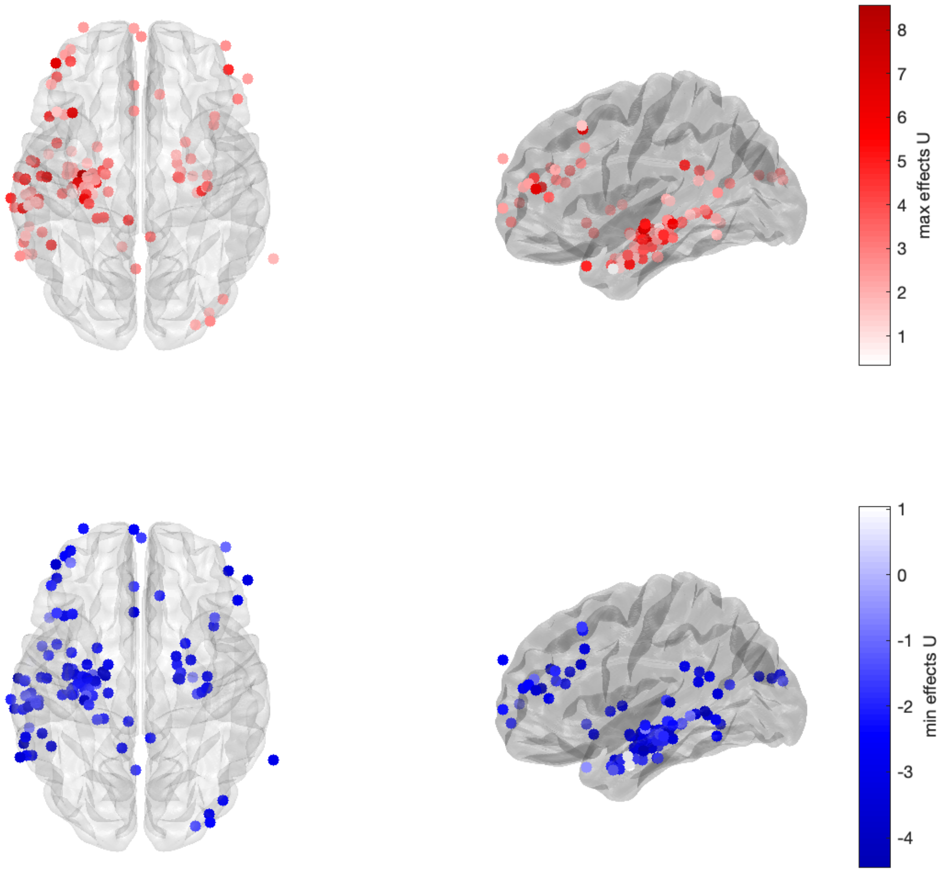

Fig. S6-2: Responding locations with max and min effect –Delta band

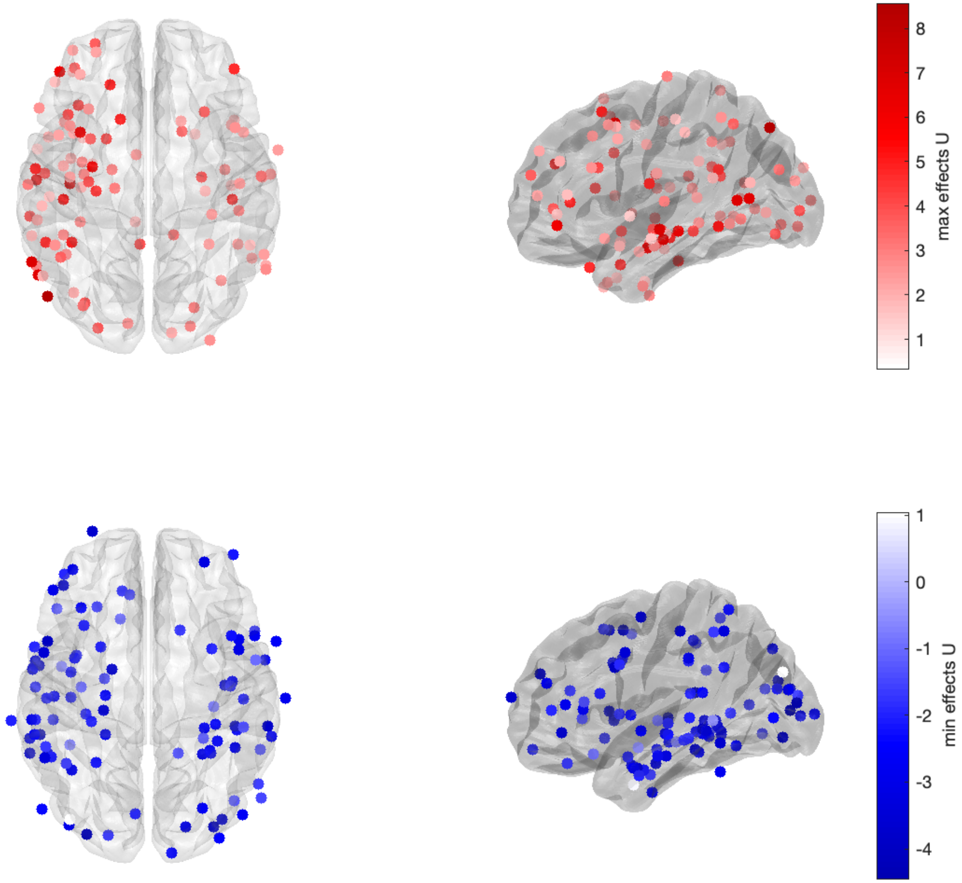

Fig. S6-3: All stim locations – Colours indicate the maxima and minima effects elicited (in other locations) by the stimulation - Theta band

Locations marked are the midpoints between anode and cathode

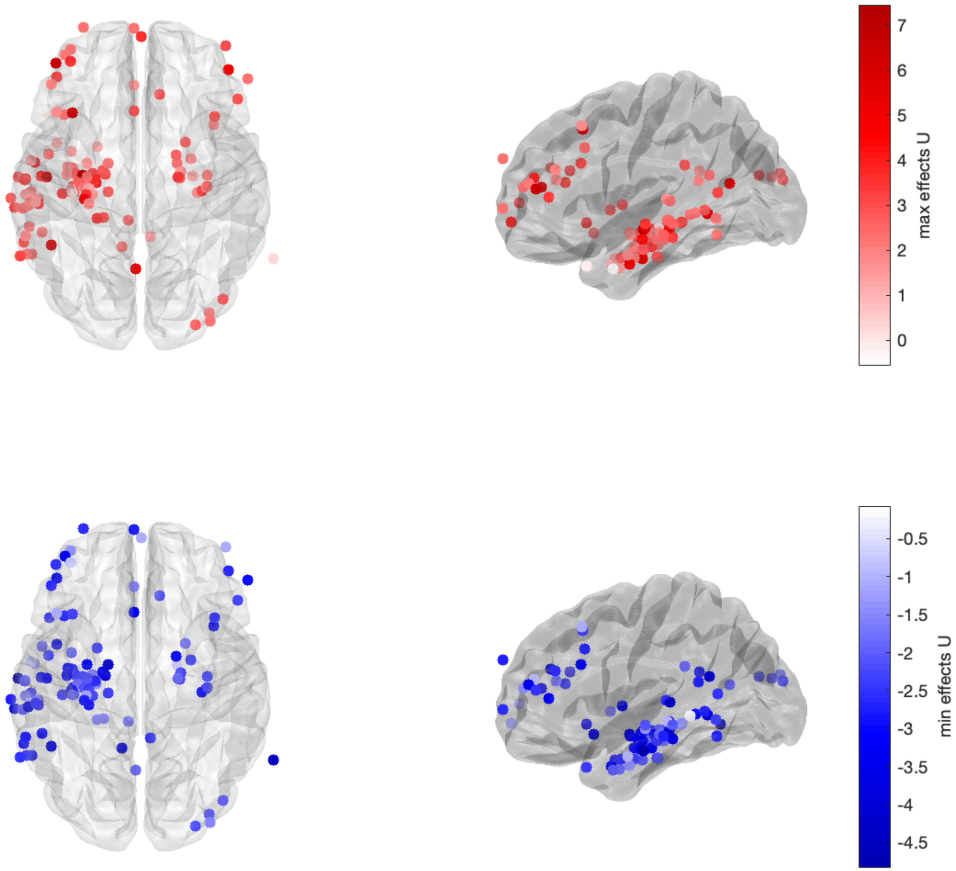

Fig. S6-4: Responding locations with max and min effect - Theta band

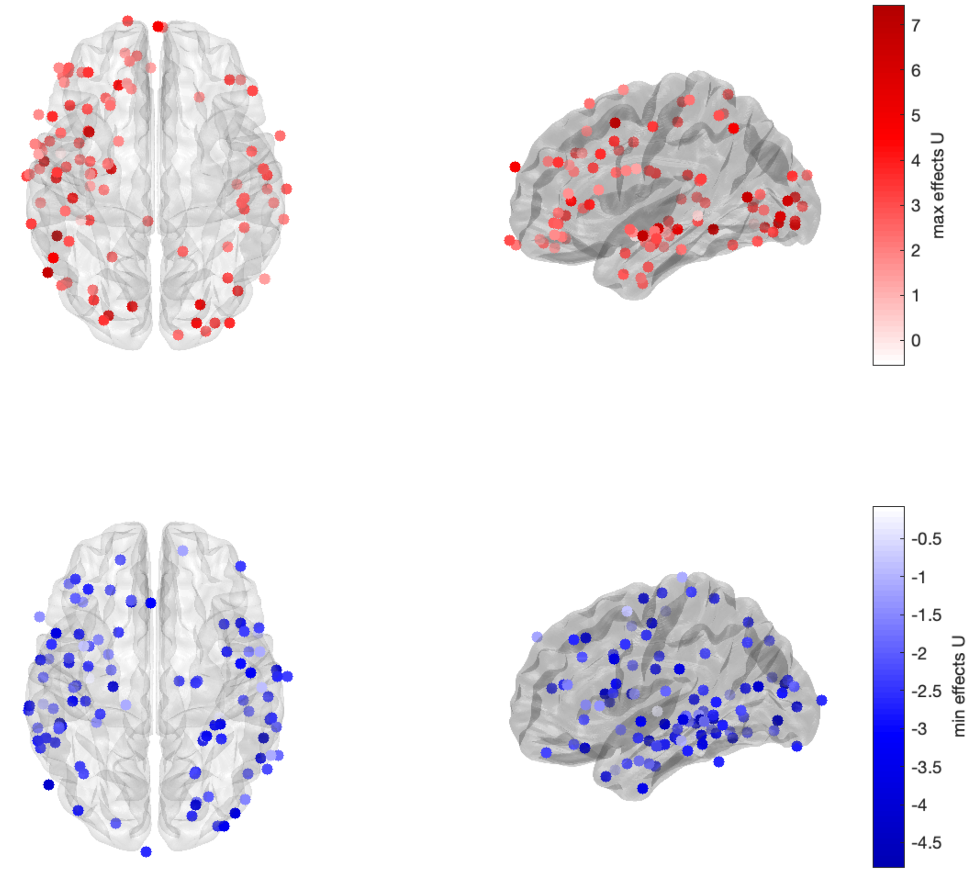

Fig. S6-5: All stim locations – Colours indicate the maxima and minima effects elicited (in other locations) by the stimulation - Alpha band

Locations marked are the midpoints between anode and cathode

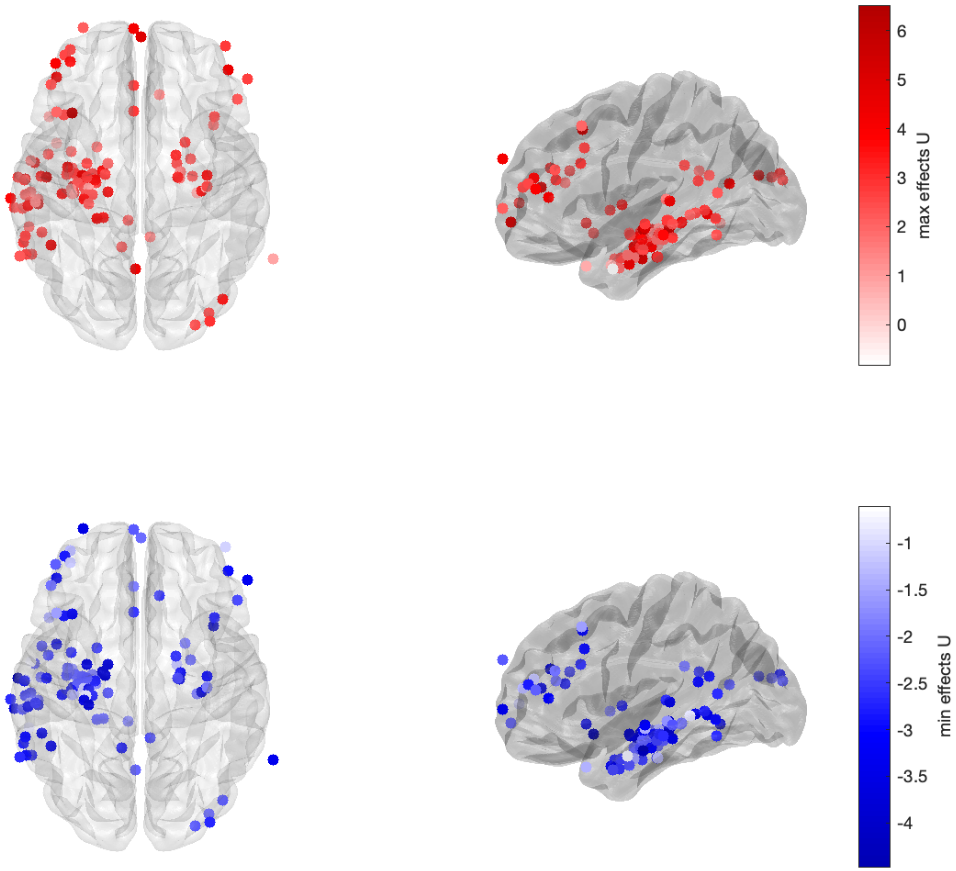

Fig. S6-6: Responding locations with max and min effect - Alpha band

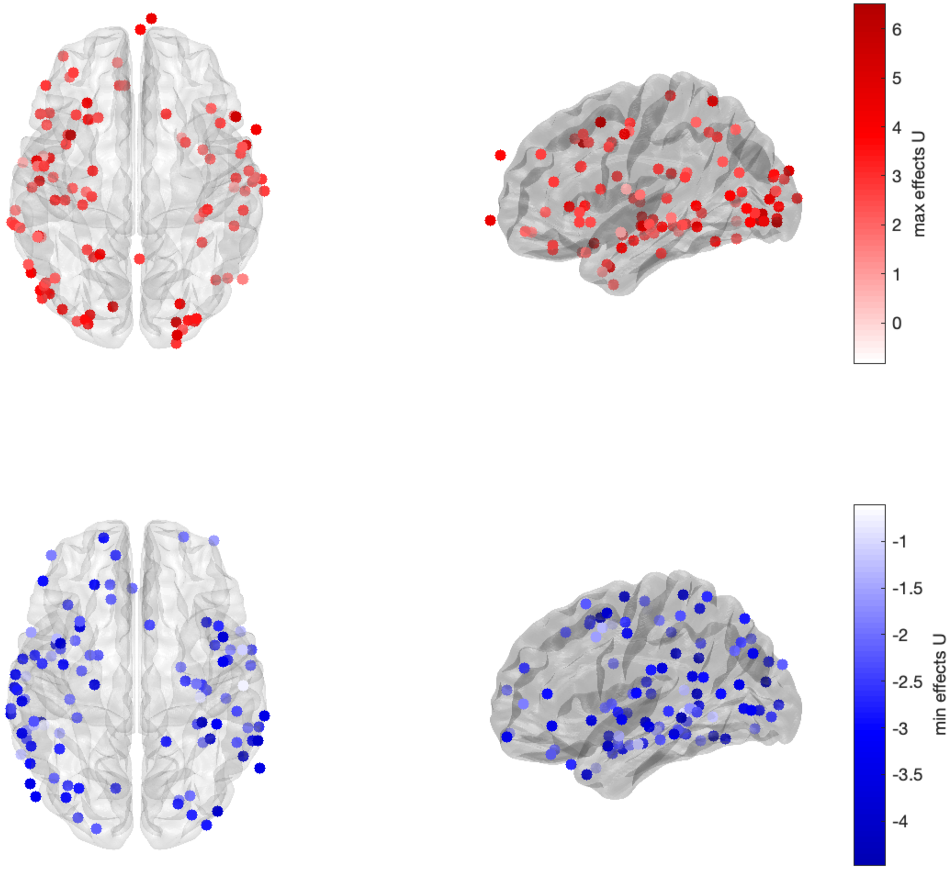

Fig. S6-7: All stim locations – Colours indicate the maxima and minima effects elicited (in other locations) by the stimulation - Beta band

Locations marked are the midpoints between anode and cathode

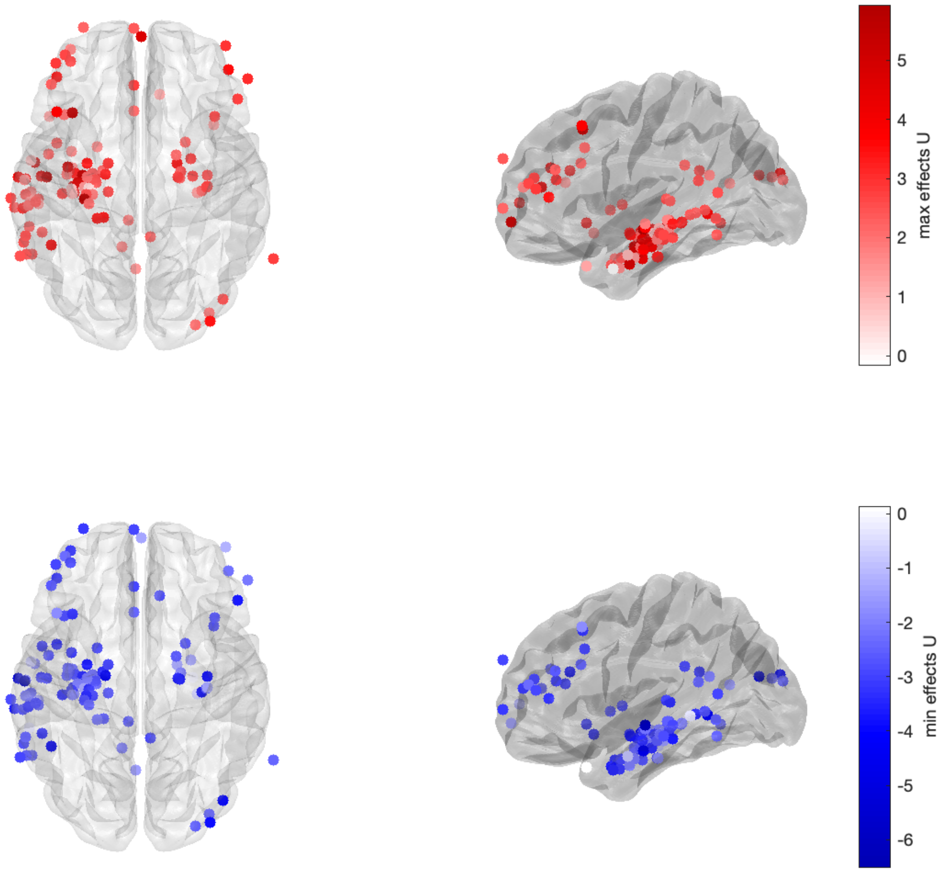

Fig. S6-8: Responding locations with max and min effect - Beta band

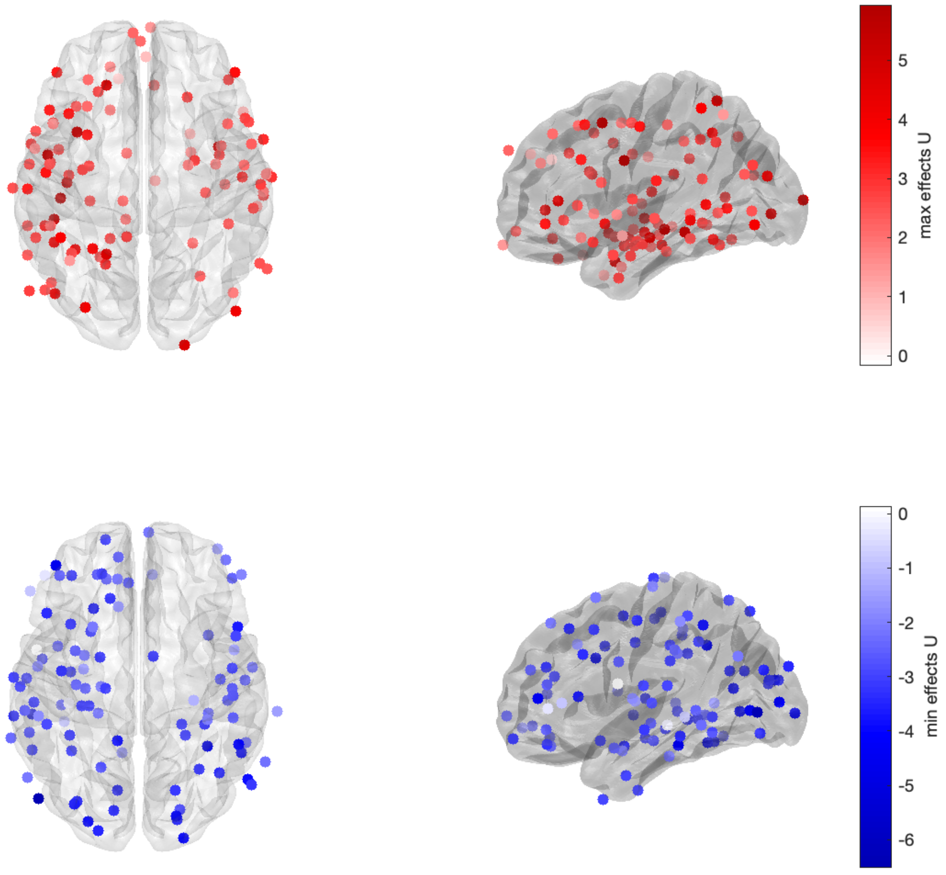

Fig. S6-9: All stim locations – Colours indicate the maxima and minima effects elicited (in other locations) by the stimulation - Gamma band

Locations marked are the midpoints between anode and cathode

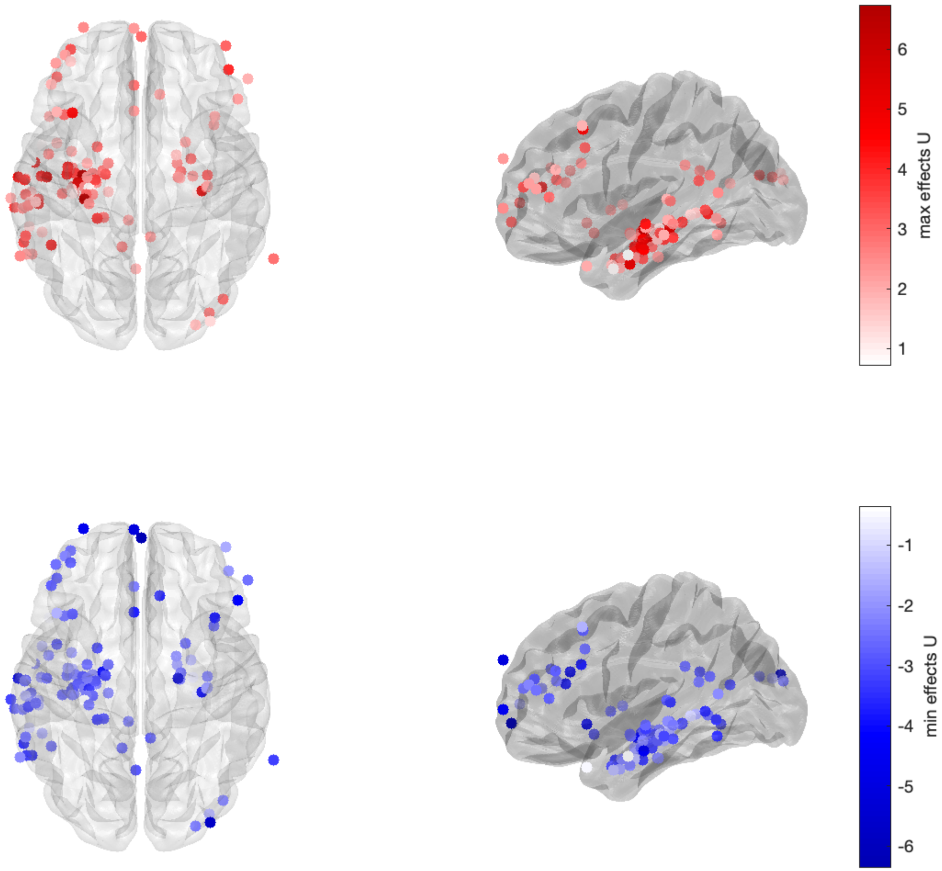

Fig. S6-10: Responding locations with max and min effect - Gamma band

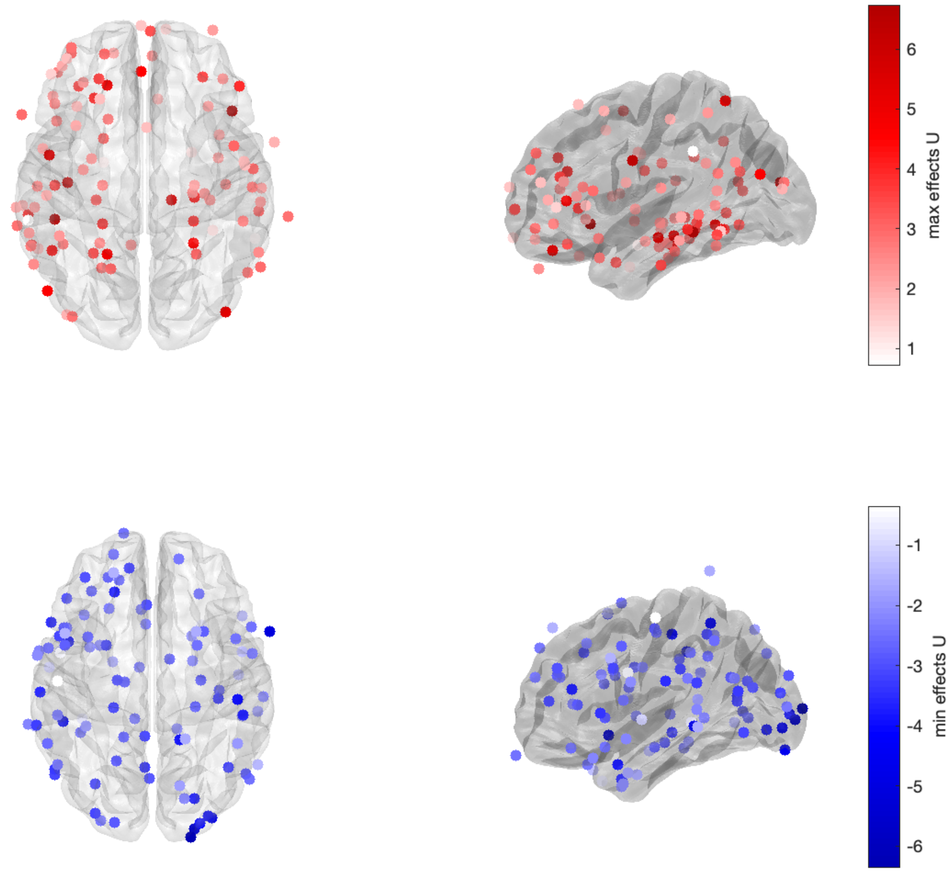

Fig. S6-11: Stim locations with at least 1 session pair – Colours indicate the averaged (across multiple session pairs) maximum consistency coefficient

Locations marked are the midpoints between anode and cathode

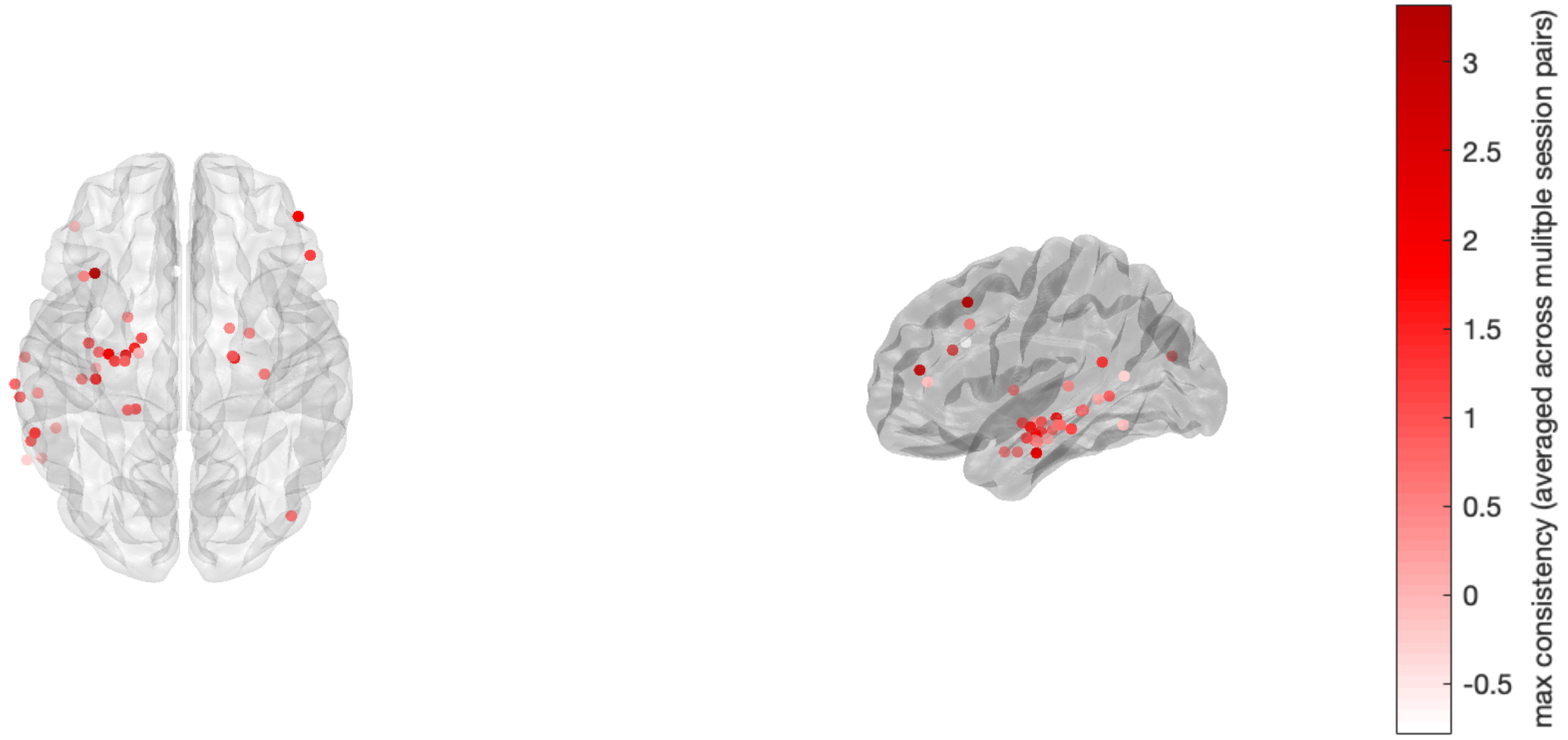

Fig S7-1: No correlation between the stimulation frequency and the max effect but there is anti-correlation between stimulation frequency and min effect across all subjects (considering all bands)

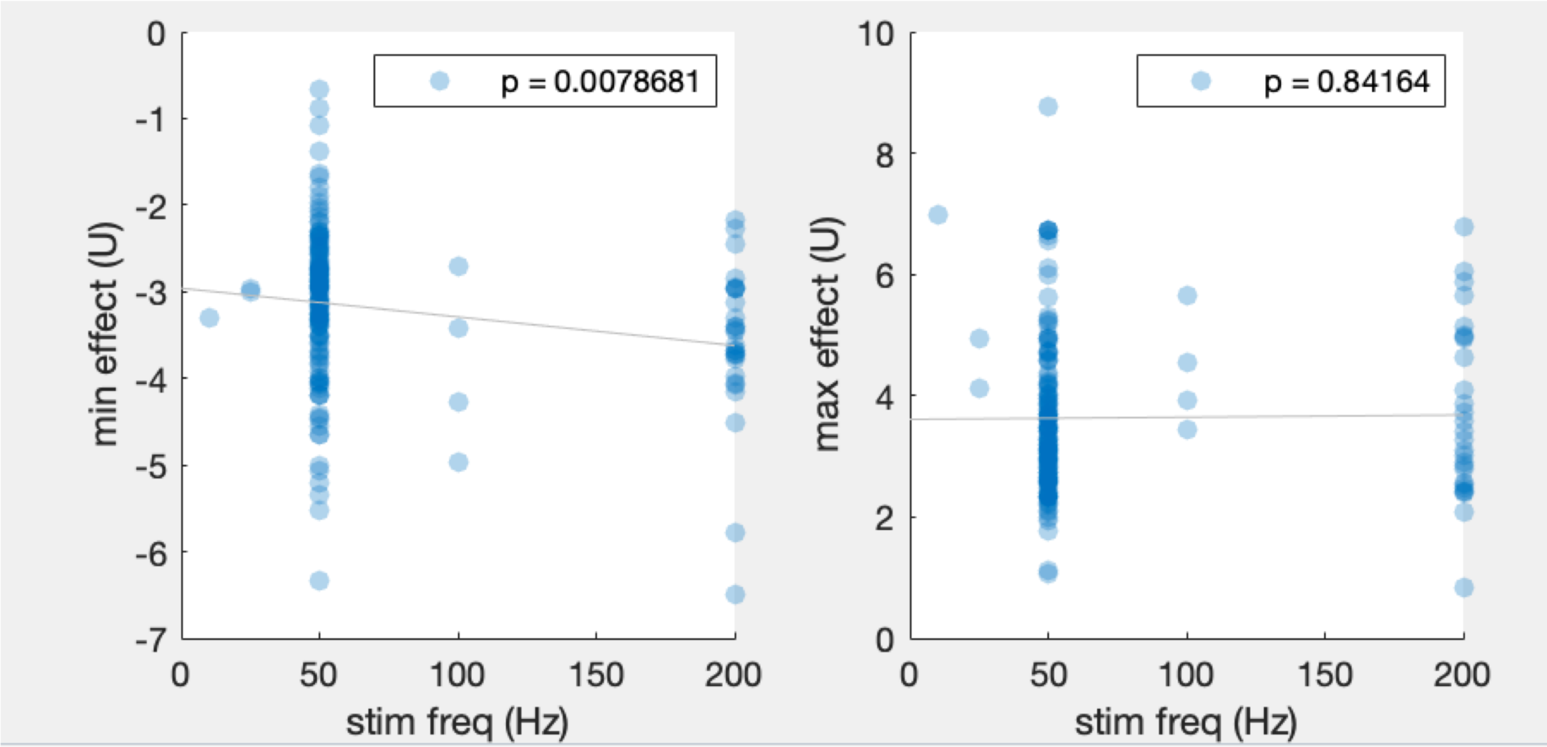

Fig S7-2: No correlation between the stimulation frequency and the min/max effect (considering delta band only)

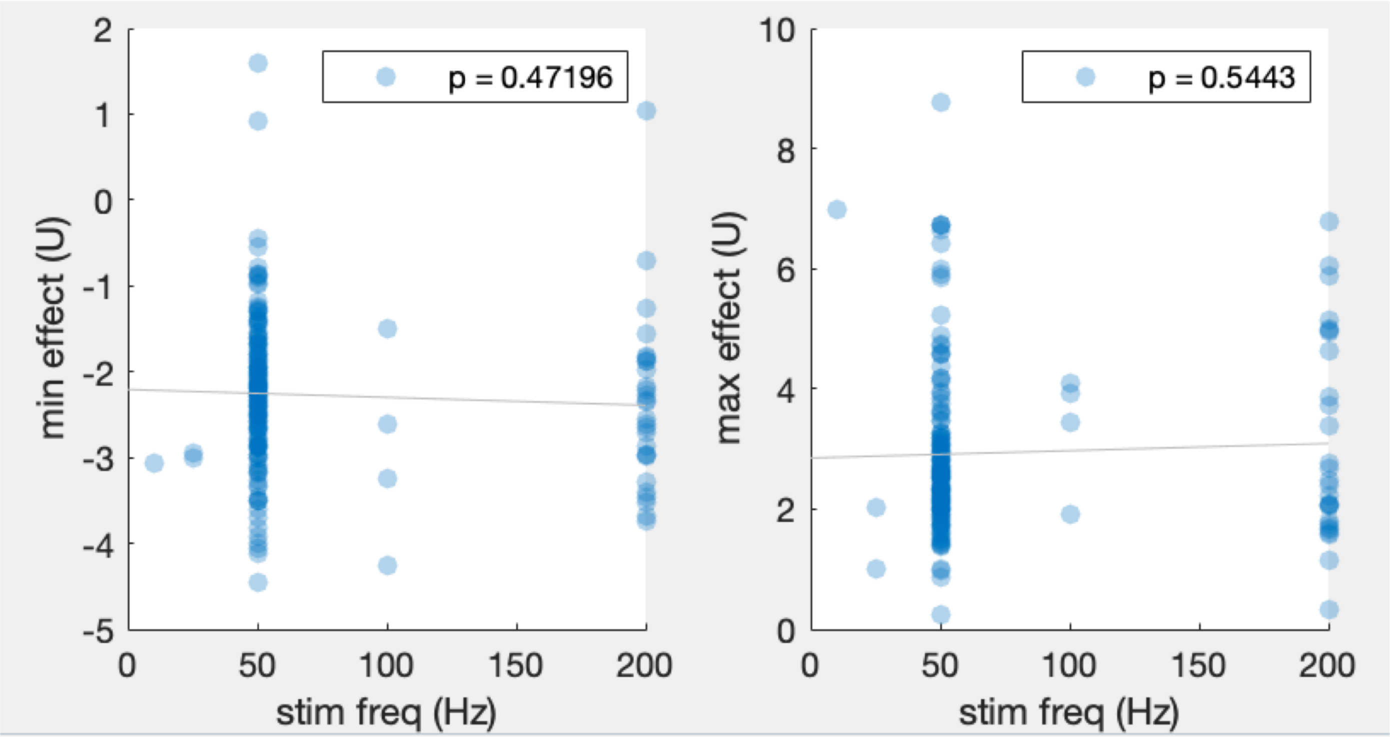

Fig S7-3: No correlation between the stimulation frequency and the max effect but there is anti-correlation between stimulation frequency and min effect (considering theta band only)

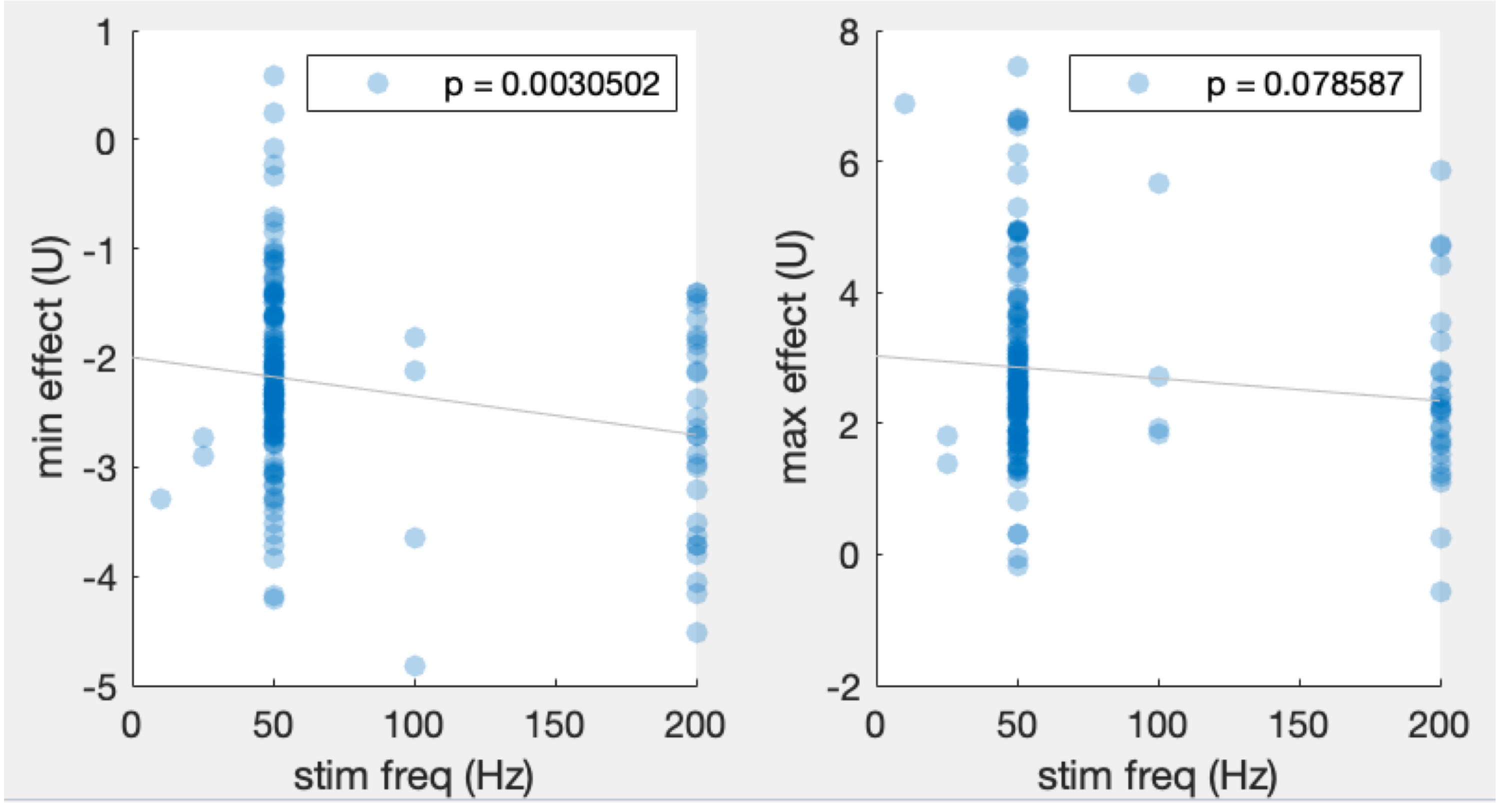

Fig S7-4: No correlation between the stimulation frequency and the max effect but there is anti-correlation between stimulation frequency and min effect (considering alpha band only)

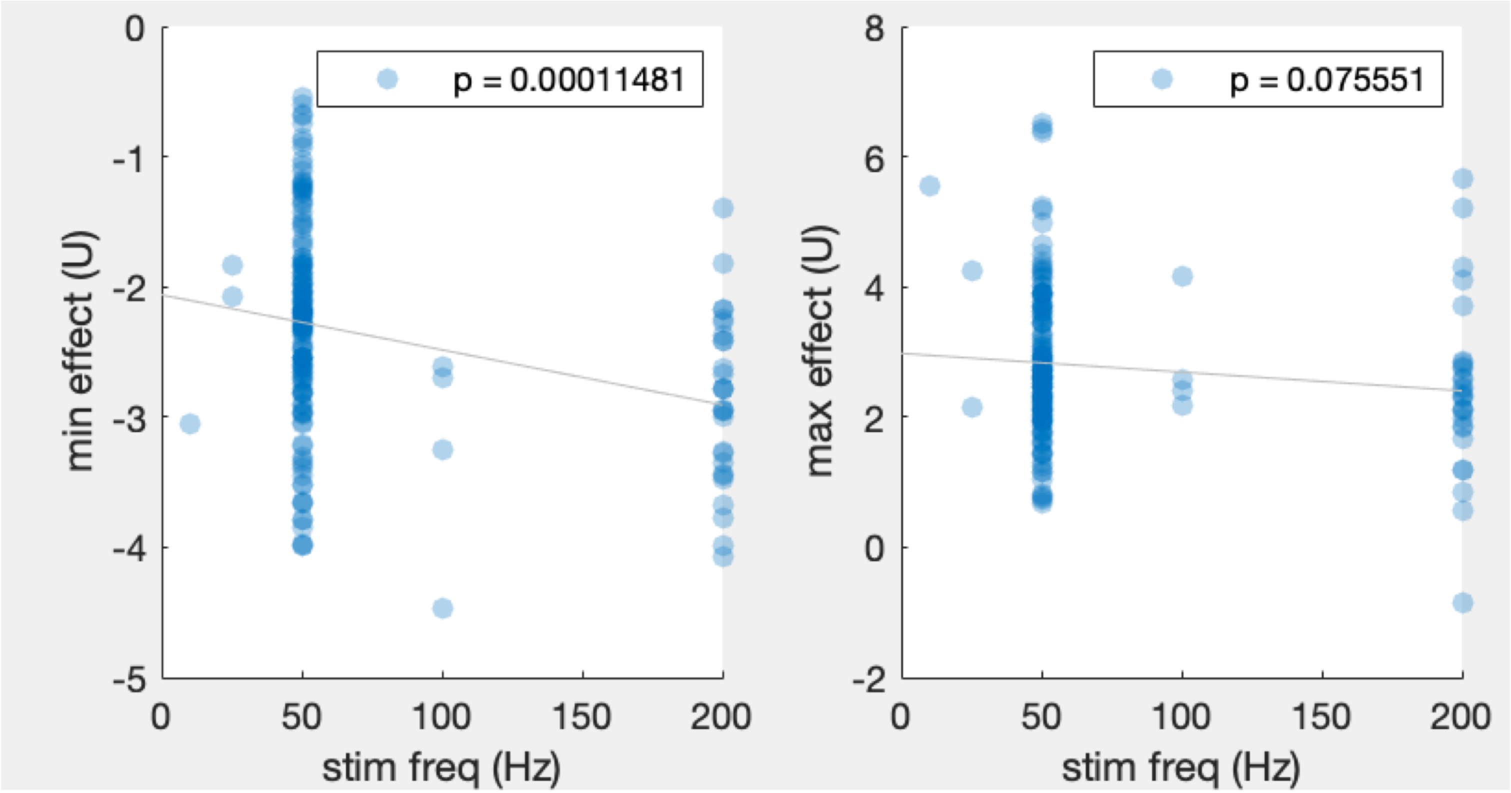

Fig S7-5: No correlation between the stimulation frequency and the max effect but there is anti-correlation between stimulation frequency and min effect (considering beta band only)

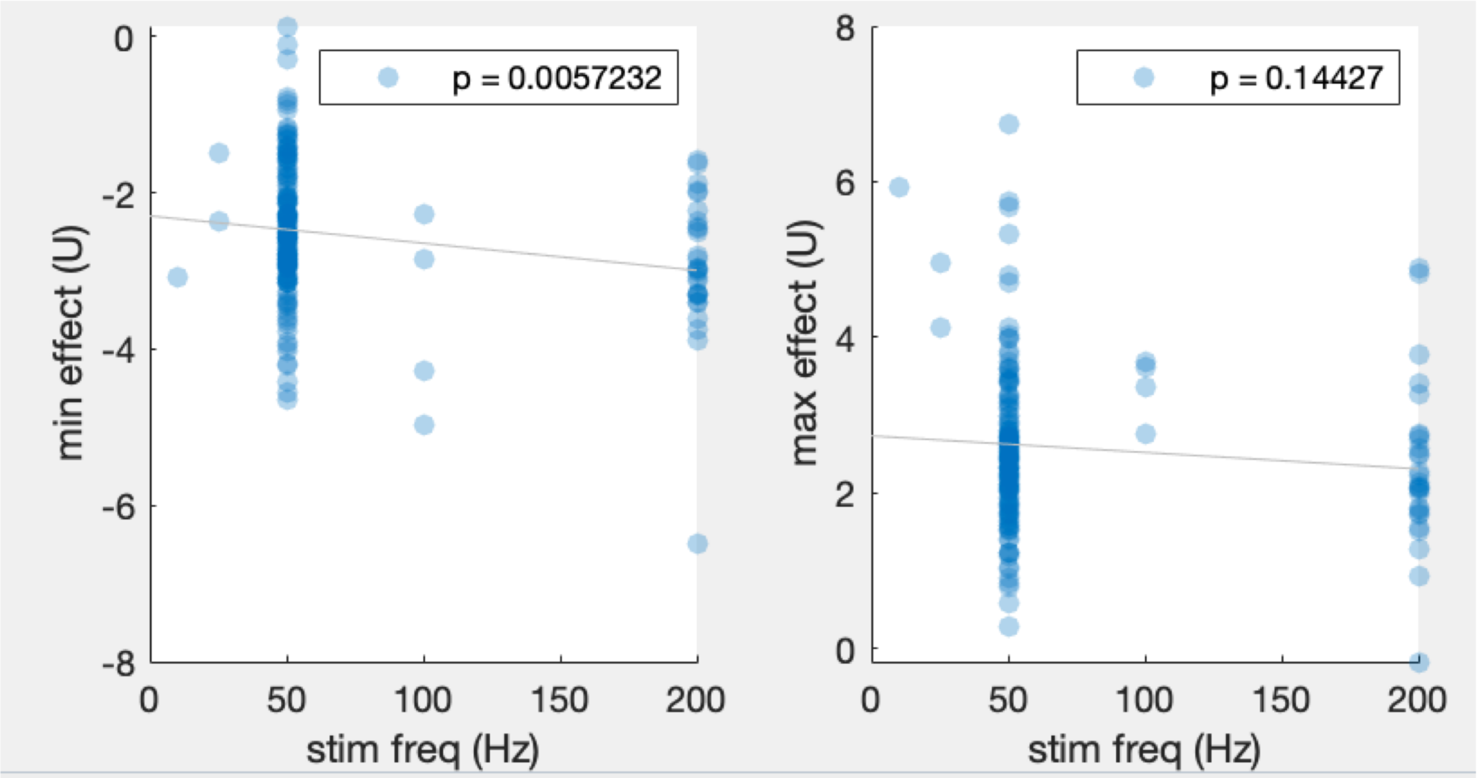

Fig S7-6: No correlation between the stimulation frequency and the min/max effect (considering gamma band only)

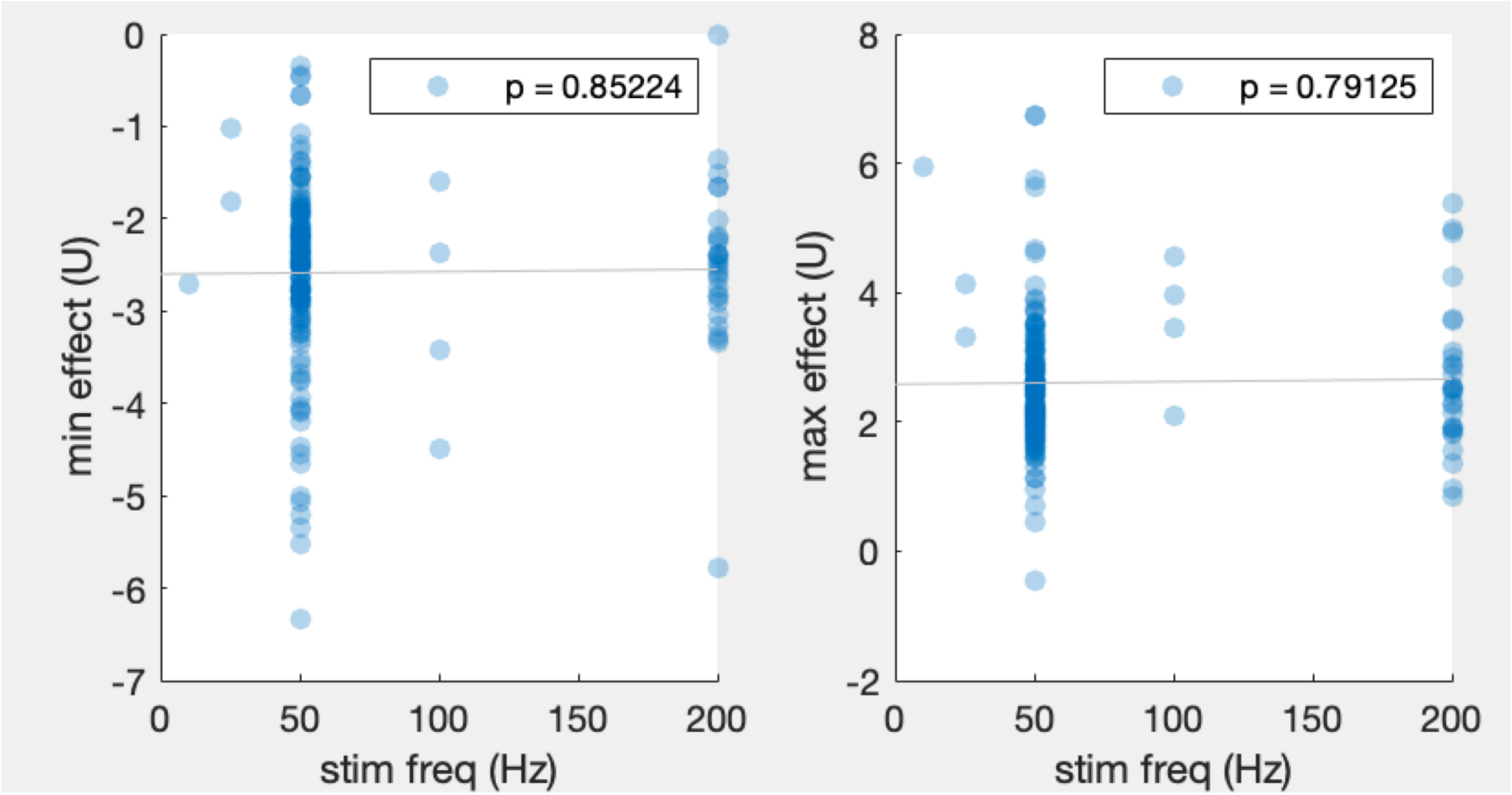

Fig S8: test for the influence of stimulation duration on the min/max effect values (Wilcoxon rank sum test, two groups of duration: short vs long).

effect on band	test on min value (p-value, z-stat)	test on max value (p-value, z-stat)
all	**0.0118, -2.5191**	**0.0163, 2.4031**
delta	0.4301, -0.7890	0.2158, 1.2377
theta	**0.0287, -2.1883**	0.1283, -1.5209
alpha	**0.0044, -2.8461**	0.5021, -0.6711
beta	0.0632, -1.8575	0.6171, 0.5000
gamma	0.7179, 0.3612	0.1217, 1.5476

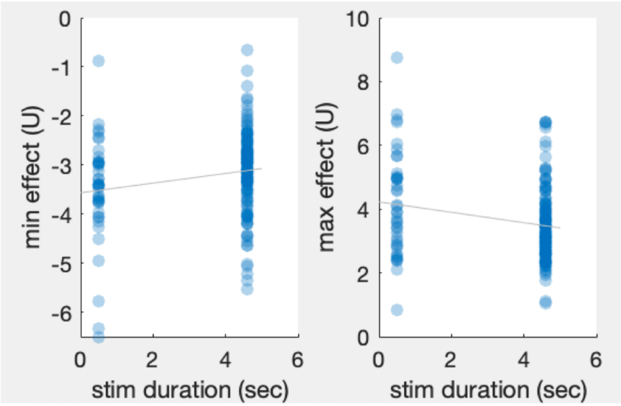

The min effect is higher (less negative) and the max effect is lower with long duration, that is, both min and max effect values tend to get closer to 0 with long stimulation

Notice that when the stimulation duration is long (4.6s) the frequency is always low (50Hz). This association between duration and frequency and the previous finding on the influence of frequency (Fig. S7) can explain the finding here, at least the relationship between min effect and duration. Further investigation on the influence of duration on consistency revealed no relation (p=0.538, Wilcoxon rank sum test on the two unpaired groups, short vs long duration), as expected since there is no relation between stim frequency and consistency.
